# A baculovirus-conjugated mimotope vaccine targeting *Mycobacterium tuberculosis* lipoarabinomannan

**DOI:** 10.1371/journal.pone.0185945

**Published:** 2017-10-05

**Authors:** Hyun-Jin Shin, Luis H. Franco, Vidhya R. Nair, Angela C. Collins, Michael U. Shiloh

**Affiliations:** 1 Department of Internal Medicine, University of Texas Southwestern Medical Center, Dallas, TX, United States of America; 2 Center for Autophagy Research, University of Texas Southwestern Medical Center, Dallas, TX, United States of America; 3 Department of Microbiology, University of Texas Southwestern Medical Center, Dallas, TX, United States of America; Emory University, UNITED STATES

## Abstract

*Mycobacterium tuberculosis*, the causative agent of tuberculosis, is a major cause of morbidity and mortality worldwide. However, an effective vaccine for *M*. *tuberculosis* is lacking. We panned a phage display library using monoclonal antibodies against *M*. *tuberculosis* liporabinomannan (LAM), an important component of the *M*. *tuberculosis* cell wall, and identified two peptide sequences, HSFKWLDSPRLR or SGVYKVAYDWQH, with high antibody affinity after multiple rounds of panning. Only the HSFKWLDSPRLR peptide induced an anti-LAM response when conjugated to either keyhole limpet hemocyanin (KLH) or to the baculovirus Autographa californica multicapsid nucleopolyherovirus (AcMNPV) when introduced into mice by injection or via intranasal inoculation, respectively. Vaccination with AcMNPV conjugated HSFKWLDSPRLR peptide delayed mortality in a mouse model of tuberculosis. Thus, we report a proof of principle *M*. *tuberculosis* vaccination strategy combining an anti-LAM mimotope with a baculovirus delivery system.

## Introduction

The clinical, social, and economic burden of tuberculosis remains high despite the recent World Health Organization (WHO) tuberculosis report indicating that progress has been made towards the global reduction of tuberculosis [1]. About one-third of the world’s population is infected with *Mycobacterium tuberculosis* while nearly 9 million cases of active tuberculosis are reported annually. In 2015 there were an estimated 10.4 million new tuberculosis cases and 1.8 million tuberculosis deaths overall, including 1.0 million cases and 170,000 deaths among children [2].

To date, the Bacillus Calmette Guerin (BCG) vaccine remains the only licensed vaccine for the prevention of tuberculosis. BCG vaccine is primarily administered in geographic areas where tuberculosis is endemic, as the protection afforded by BCG vaccination is limited to reducing the incidence of disseminated tuberculosis in children [3, 4]. BCG neither prevents initial infection in children nor provides any significant protection in adults [3, 4]. To address this deficiency, several new vaccines are in various stages of development [5]. Although multiple innovative approaches have been proposed and tried, progress has been slow, and only one major candidate has completed a Phase IIb efficacy trial with disappointing results [6]. Thus, there remains an urgent need to develop novel vaccination strategies for *M*. *tuberculosis*.

A highly successful method for vaccination against both bacterial and viral pathogens has been to induce antibodies against critical cell surface molecules such as the capsular polysaccharide of *Streptococcus pneumonia* or the hemagglutinin of influenza. Because *M*. *tuberculosis* is enveloped by a thick carbohydrate and lipid-rich cell wall, it has not been amenable to similar vaccination strategies. Further, the prevailing paradigm in tuberculosis biology has been that antibodies play a minimal role in controlling infection since *M*. *tuberculosis* has a predominantly intracellular lifestyle. However, accumulating data suggests that anti-mycobacterial antibodies can be protective in models of *M*. *tuberculosis* infection [7–12]. Passive immunization with antibodies against either the dormancy protein alpha-crystallin [13, 14] or the cell wall component lipoarabinomannan (LAM) [15] is protective following an aerosol challenge in mice. Since *M*. *tuberculosis* infection occurs via an aerosol route, an ideal vaccine would generate a robust immunologic response in the airway, perhaps by utilizing a mucosal vaccination strategy [16]. Moreover, such a vaccine should be inexpensive and easily delivered in resource-limited settings.

Recently, there has been significant interest in using adenovirus as a vaccine vector for *M*. *tuberculosis* antigens [17]. However, the host immune response to adenovirus has limited this approach [18, 19]. To that end, the baculovirus Autographa californica multicapsid nucleopolyherovirus (AcMNPV) may serve as an improved vaccine vector as it can be delivered via an aerosol route to effectively transduce mammalian cells [20, 21]. Importantly, a very useful characteristic of AcMNPV is that it naturally infects insects but does not cause disease in mammals, as it replicates poorly within mammalian cells [21]. After aerosol infection, AcMNPV enters the cytoplasm of macrophages and dendritic cells, thus eliciting both humoral and cell mediated immunity characterized by a vigorous Th1 response [22–24]. To date, AcMNPV has been used to successfully vaccinate against influenza [22] and to generate a robust response against a malaria protein [25]. In the case of influenza, when recombinant AcMNPV expressing full-length influenza hemagglutinin was used to inoculate mice intranasally, mice were protected from lethal challenge with influenza due to immunoglobulin production and stimulation of a potent innate and cell mediated immune response [22]. Likewise, a recombinant baculovirus that both displayed the malaria circumsporozoite protein on its surface and drove intracellular expression of the protein within antigen presenting cells induced antibody, CD4 and CD8 responses in vitro [25]. Most animals lack pre-existing antibodies against baculovirus, and baculovirus is stable at room temperature, which could eventually allow it to be utilized as a human vaccine without need for refrigeration.

The mycobacterial cell wall is a protein, lipid and carbohydrate rich structure thought to be involved in the virulence of *M*. *tuberculosis*. The major constituents are the mycolic acid lipids, arabinomannan (AM) and lipoarabinomannan (LAM) polysaccharides, and cell wall associated proteins [26]. Previously, when various components of the cell wall have been delivered subcutaneously, vaccination has been equivalent to the live attenuated *Mycobacterium bovis* bacillus Calmette-Guerin (BCG) vaccine. However, direct vaccination with lipids or cell wall sugars may be deleterious, as they have potent immunomodulatory activities. An alternative to using immunomodulatory lipids or sugars is to utilize mimotopes, short peptides that can elicit protective antibodies against non-peptide antigens [27]. Such mimotopes behave as surrogate antigens, inducing an immune response that is cross-reactive with the native antigen. Peptide mimotopes have been used successfully to vaccinate against the *Cryptococcus neoformans* capsular polysaccharide [28], and importantly, mimotopes targeting *M*. *tuberculosis* LAM have been reported [29–31]. However, the ability of mimotopes to protect against *M*. *tuberculosis* infection has not been tested to date. Here, we discover novel mimotope peptides for *M*. *tuberculosis* LAM and establish that conjugation to baculovirus can lead to a protective immune response.

## Materials and methods

### LAM antibodies and screening

Anti-LAM mouse monoclonal antibodies specific either for non-mannose-capped LAM, CS35 (Antibody class IgG_3_κ; NR-13811), or mannose-capped LAM (ManLAM), CS40 (Antibody class IgG1κ; NR-13812) and rabbit polyclonal rabbit anti-Mtb LAM antiserum (NR-13821) were generously provided by BEI Resources NIAID, NIH. To compare binding reactions of these antibodies with LAM, we performed ELISAs with LAM as the coating antigen. Five μg of LAM (NR-14848; BEI Resources NIAID, NIH) was used to coat each well of a 96 well plate (Greiner Bio-One Catalogue #655–001) following classic ELISA methods. Secondary anti-mouse and anti-rabbit antibodies conjugated to HRP were from Jackson ImmunoResearch Laboratories Inc. (catalogue # 715-035-151 and 111-035-003, respectively). Detection of bound antibody complexes was with the 1-Step^TM^ Ultra TMB-ELISA substrate solution (Thermo Scientific Co, Ltd; catalogue #34028).

### Screening of phage displayed peptide library

The twelve-mer phage display peptide library (New England Biolabs; E8110S) was panned with the selected mAb and titrated by plaque assay according to manufacturer’s instructions. Briefly, a well of a 96-well microtiter plate was sensitized with 25μg of selected mAb and incubated at 4°C overnight. Blocking was performed with 350 μl of BSA (5mg/ml) in 50 mM Tris-buffered saline (TBS) for 1 hour at room temperature followed by six washes with 50mM TBS containing 0.1% Tween 20 (0.1% TBS-T) to remove excess antibody and blocking reagent. Approximately 2×10^11^ pfu recombinant phage were added to the well and incubated for 1 hour. Unbound phage were removed by repeat (10X) washing with 0.1% TBS-T. Antibody-bound phage were eluted by treatment with 100 μl of 0.2M Glycine-HCl (pH 2.2) followed by 15 μl of 1M Tris-Cl (pH 9.1) containing BSA. The eluted phage were amplified in log phase *E*. *coli* ER2738 and harvested in the culture supernatant at 12,000 × *g* for 10 minutes and concentrated by PEG/NaCl precipitation. Bio-panning was repeated 4 times on amplified phage eluate using a wash buffer containing 0.5% Tween 20 (0.5% TBS-T) to enrich the pool of phage with strong binding affinity to the mAb. Eluted and amplified phages were titrated after each round of panning.

### Sequencing of mAb-specific phage

Following manufacturer’s instructions, 10 phage colonies recovered after the fifth panning step were randomly selected and amplified. Each selected phage was excised from the bacterized agar and cultured in *E*. *coli* ER2738. Phage genomic DNA was extracted by treatment with iodide buffer (pH 8.0) and purified by ethanol precipitation. The phage heptapeptide-gIII fusion gene was then sequenced and the corresponding amino acid sequence of the 12-mer peptide was deduced from the resulting nucleotide sequence using the reduced genetic code chart.

### Peptide synthesis

Peptides were synthesized by the UTSW peptide synthesis core and are summarized in Table 1. All peptides were synthesized with an N-terminal cysteine residue to permit chemical conjugation to KLH and baculovirus. Synthetic peptides were dissolved in distilled water and kept at -20°C until use.

**Table 1:** Mimotope peptides used in this study.

Peptide	Sequence	Reference
P1	CQEPLMGTVPIRAGGGS	[29]
P1 scramble	CQLGAIEMPGSPGTVGR	This work
P1 biotin	CQEPLMGTVPIRAGGGSR-Biotin	This work
P4	CMSPRATI	[30]
P4 scramble	CIPARMTS	This work
P6	CSHRLLQTYWSSA	[30]
P6 scramble	CQSTSHLYASWLR	This work
P8	CISLTEWSMWYRH	[31]
P8 scramble	CRSWEWHSTLMYI	This work
HS	CHSFKWLDSPRLR	This work
HS scramble	CPHDFRLWSLSRK	This work
SG	CSGVYKVAYDWQH	This work
SG scramble	CGSYVVAYWDKHQ	This work

### Peptide ELISA binding assay

ELISA plate wells were coated with each peptide (2 μg in 0.1M NaHCO_3_, pH 8.6) and incubating overnight at 4°C. After overnight incubation, plates were blocked with BSA (5mg/ml) for 1 hour at 4°C. For controls we coated wells with equal amounts of LAM or BSA. After washing with TBST, diluted anti-LAM antibodies were added to the ELISA plate and incubated at room temperature for 1 hour. Plates were washed with TBST and HRP-conjugated secondary antibodies were added and incubated for 1 hour. After washing 3 times with TBST, 50 μl of the 1-Step^TM^ Ultra TMB-ELISA (Thermo Scientific Co, Ltd) substrates was added in the dark. After color development, 50 μl of 2M sulfuric acid was added to stop the reaction and absorbance was measured at 450 nm.

### KLH conjugation

Peptide conjugation to KLH was performed using the Imject Maleimide Activated Carrier Protein Kit (Thermo Scientific Co, Ltd; catalogue #77115) following the manufacturer’s instructions.

### Baculovirus conjugation

We obtained wild type AcMNPV (5 x 10^8^ PFU/ml) from Kinnakeet Biotechnology (USA). Conjugation of target peptides to AcMNPV was performed using the Sulfo-SMCC (sulfosuccinimidyl 4-(N-maleimidomethyl)cyclohexane-1-carboxylate) reagent (Thermo Scientific catalogue #22322) following manufacturer’s instructions as described [23]. Briefly, 4 mg/ml of Sulfo-SMCC crosslinking reagent was added to 10 mg AcMNPV in conjugation buffer and incubated for 30 minutes at room temperature. After desalting to remove unbound crosslinker, 10 μg of peptide was added to the SMCC crosslinker-coated AcMNPV and incubated for 20 minutes at room temperature followed by a desalting step to remove unbound peptide. The AcMNPV-peptide conjugates were then stored at -80°C until use.

### Mouse vaccination to measure antibody responses

Swiss Webster outbred mice (5 mice/group, Taconic Biosciences) housed under specific pathogen free conditions were vaccinated with PBS or mimotopes-conjugated to KLH or baculovirus. Animals were randomly assigned to experimental groups at the start of the experiment. For mice receiving PBS, KLH alone or peptide-conjugates to KLH, inoculations were via subcutaneous injection. For the baculovirus groups, inoculations were intranasal. On day 0, serum from each mouse was collected from the retroorbital plexus (pre-immune), and 10 μg of peptide-conjugate or control was inoculated per animal. When testing mimotopes conjugated to KLH, vaccination also included alum adjuvant. Likewise, when testing if anti-LAM antibodies generated in mice could react against mimotopes, Swiss Webster outbred mice were vaccinated with LAM with alum. Animals were boosted at two week intervals for 3 additional times. Before each boost, serum was collected from each mouse and stored at -20°C until use.

### Mouse serology

To perform ELISAS, 96-well ELISA plates (Greiner Bio-One Catalogue #655–001) were coated with 10 μg of corresponding coating antigens: HS peptide, HSSR peptide, Baculovirus, SG peptide, SGSR peptide, or LAM resuspended in coating buffer (0.1M NaHCO_3_, pH 8.6). After overnight coating at 4°C, blocking (5% skim milk in PBS) was performed for 1 hour at room temperature. After washing 3 times with washing buffer (0.01% Tween-20 in PBS), mouse serum was added to the appropriate wells and binding proceeded at room temperature for 1 hour. Plates were washed 3 times with washing buffer (0.01% Tween-20 in PBS) and HRP-conjugated anti-mouse IgG at 1:5,000 dilution (Jackson ImmunoResearch Laboratories Inc. catalogue # 715-035-151) was added to each well. After incubating for 1 hour at room temperature, the plate was washed 3 times with TBST and 50 μl of the 1-Step^TM^ Ultra TMB-ELISA substrate (Thermo Scientific) was added. Reactions were stopped by the addition of 50 μl of 2M sulfuric acid and absorbance was measured at 450nm.

### Mouse aerosol infection and survival study

To test the ability of the anti-LAM mimotope to protect mice from subsequent tuberculosis infection when conjugated to baculovirus, outbred Swiss Webster mice (n = 15 per group) housed under specific pathogen free conditions were vaccinated according to the protocol described above. Animals were randomly assigned to experimental groups at the start of the experiment. Mice receiving BCG Pasteur were vaccinated once subcutaneously (s.c.) with 10^6^ bacteria in 0.2 ml PBS 8 weeks prior to receiving an *M*. *tuberculosis* Erdman challenge. After either baculovirus or BCG vaccination, mice were infected with ~200 CFU of *M*. *tuberculosis* Erdman strain in a Glas-Col aerosolization chamber as described [32–34]. On day 0, 5 unvaccinated mice were euthanized using CO_2_ inhalation following 2013 AVMA guidelines to determine the initial CFU. On day 30, 5 mice per group were euthanized using CO_2_ inhalation, their organs (lungs, mediastinal lymph nodes, liver and spleen) harvested and CFU determined for each organ by dilution and plating. Mice in the survival study were monitored weekly for weight loss and were euthanized using CO_2_ inhalation following 2013 AVMA guidelines when they had lost >15% of their body weight, as previously established [32, 34–36]. No animals showed severe signs of illness following infection and no animals died (without euthanasia) due to the experimental procedures.

### Statistical analysis

Statistical analysis was performed using GraphPad Prism version 6.01. Two-tailed unpaired Student’s *t* test was used for single comparisons. Analysis of Variance (ANOVA) was used for experiments with multiple comparisons using Holm-Sidak’s multiple comparison test for individual comparisons. Gehan-Breslow-Wilcoxon test was used for mouse survival experiments.

### Ethics statement

This study was carried out in strict accordance with the recommendations in the Guide for the Care and Use of Laboratory Animals of the National Institutes of Health. The protocol was approved by the Institutional Animal Care and Use of the University of Texas Southwestern Medical Center.

## Results

### Testing existing mimotopes in vitro and in mice

**Fig 1:**
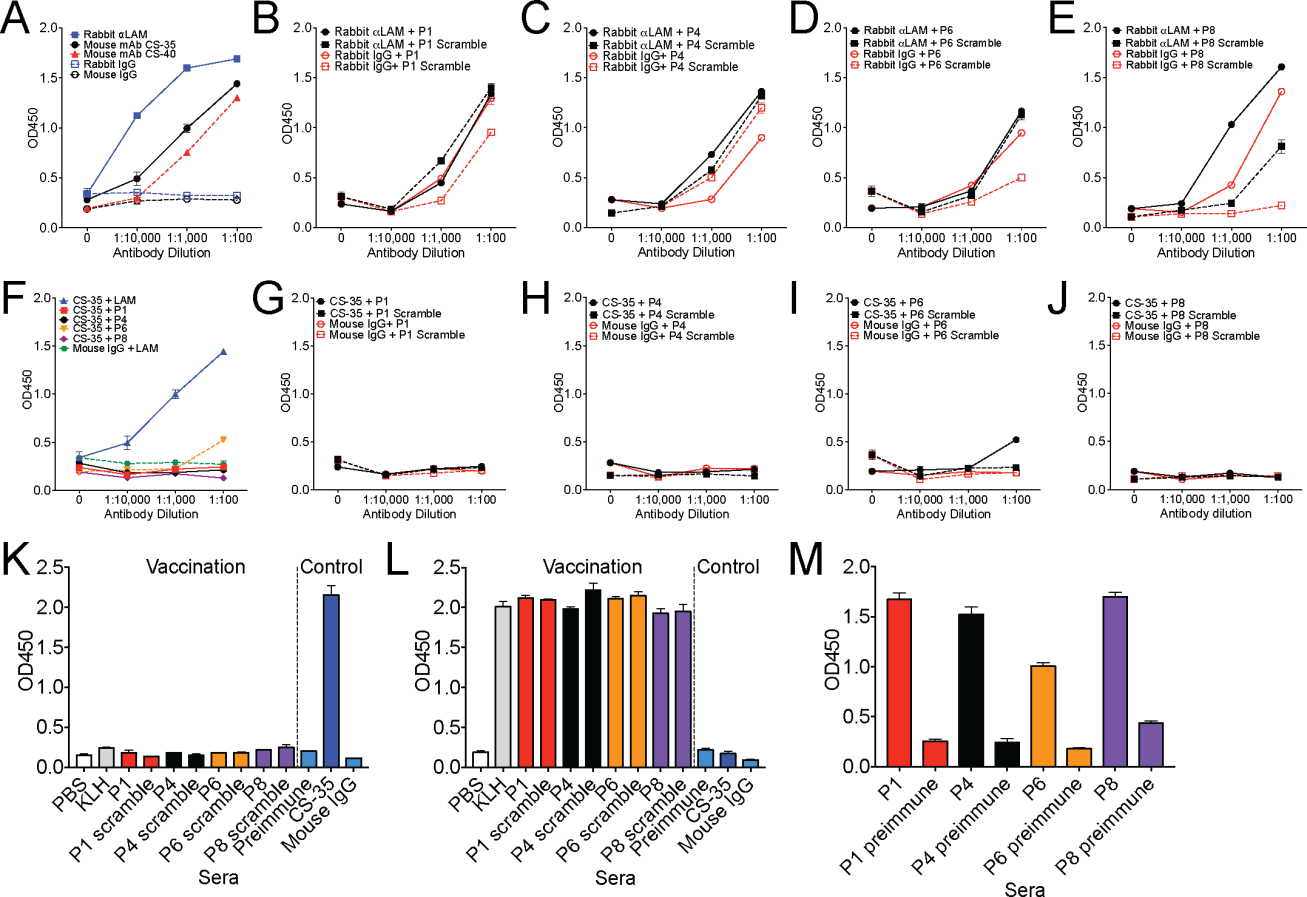
Analysis of existing LAM mimotopes. A. Testing a polyclonal rabbit and two mouse monoclonal antibodies in a LAM ELISA. B-E Mimotope peptides or their scramble controls were conjugated to KLH and mimotope behavior determined by ELISA using the rabbit polyclonal anti-LAM antibody. F-J Mimotope peptides or their scramble controls were conjugated to KLH and mimotope behavior determined by ELISA using the mouse monoclonal CS-35 anti-LAM antibody. K-M Mice (n = 5 per peptide) were vaccinated with PBS, KLH alone, or mimotope peptides or scramble controls conjugated to KLH and sera collected after 4 vaccinations. Mouse sera (1:100 dilution) was tested by ELISA against LAM (K), KLH (L) or specific peptides (M) with CS-35 as a positive control and preimmune sera or mouse IgG as negative controls. One of two representative experiments is shown.

To begin testing the ability of peptide mimotopes to induce an anti-LAM response and protect mice in vivo, we first tested either a rabbit polyclonal anti-LAM antibody or two mouse monoclonal anti-LAM antibodies (CS-35 and CS-40) for their ability to bind LAM by ELISA (Fig 1A). All three antibodies were able to bind LAM, though the rabbit polyclonal could bind at a lower titer compared to the two mouse monoclonal antibodies tested (Fig 1A). We next synthesized 4 previously published peptides and their scrambled controls (Table 1) [29–31], conjugated the peptides to KLH and tested the ability of either the rabbit anti-LAM polyclonal antibody or the mouse monoclonal antibody CS-35 to bind the KLH-conjugated peptides in vitro by ELISA (Fig 1B–1J). Of the previously published peptides, only P8 could be specifically detected by the rabbit anti-LAM polyclonal antibody above control (Fig 1E), and only at the 1:1000 dilution. Likewise, none of the peptides conjugated to KLH were bound by the CS-35 mAb (Fig 1F–1J). We next tested if the peptides could induce an anti-LAM immune response in mice. We injected outbred Swiss-Webster mice 4 times with PBS, KLH alone, KLH conjugated to peptide or KLH conjugated to control peptide along with an alum adjuvant, and we collected sera from mice after the 4^th^ injection. Using a LAM ELISA, we found that as with the in vitro assay, none of the vaccinations with KLH-conjugated mimotope peptides induced anti-LAM antibodies at an antibody dilution of 1:100, while the CS-35 mAb showed a strong response (Fig 1K). As expected, immunization induced a robust anti-KLH (Fig 1L) and anti-peptide (Fig 1M) response. Taken together, our data indicates that at least in our hands, the previously identified anti-LAM mimotopes were not bound by anti-LAM antibodies in vitro and did not induce an anti-LAM mimotope response during experimental vaccination.

### Screening phage display libraries with anti-LAM monoclonal antibodies

To identify novel mimotope sequences for LAM antibody induction, we chose to pursue biopanning of phage display libraries with monoclonal anti-LAM antibodies rather than the polyclonal anti-LAM antibody since monoclonal antibodies have defined epitope specificities as compared to polyclonal antibodies. We conducted five rounds of biopanning of a 12-mer phage display library (NEB) independently with either CS-35 or CS-40. As expected, the number of high-affinity phages recovered increased significantly over the course of the biopanning. After the fifth panning cycle, we sequenced 10 randomly selected phages per antibody. When biopanning with CS-40 mAb, 2 peptide motifs predominated: HSFKWLDSPRLR (hereafter called HS peptide after the two N-terminal amino acids) accounted for 70% (7/10) of the sequenced phages, while SGVYKVAYDWQH (hereafter called SG peptide) accounted for 30% (3/10). Biopanning with CS-35 mAb revealed the predominance of only 1 peptide motif, SGVYKVAYDWQH, which was identical to the SG peptide obtained from biopanning with CS-40. To determine if the two identified peptide motifs represented false-positive target-unrelated peptides (TUP) [37], we searched the SAROTUP [38] and Mimo-DB [39] databases with each peptide. While neither peptide was identified as a TUP using the TUPScan software, both peptides had previously been identified during other target searches. In particular, the SG peptide has been identified 5 times previously [40–44], while the HS peptide has only been identified once [40]. Interestingly, both the SG and HS peptide were identified in the same screen for peptides that bind the calcium-independent mannose-6-phosphate receptor [40].

### Characterization of mimotope behavior of identified peptides

**Fig 2:**
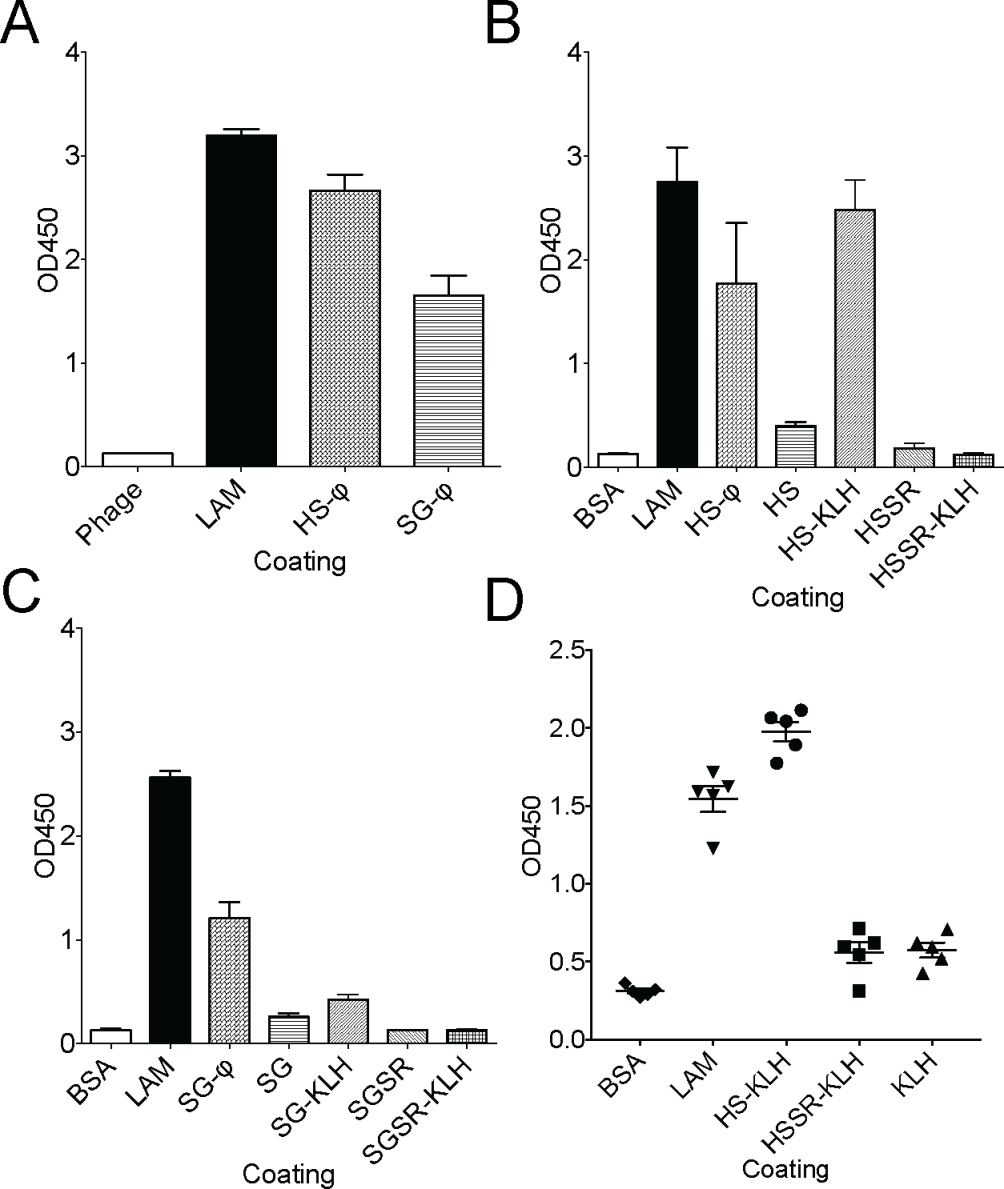
Analysis of novel anti-LAM mimotopes by ELISA. A. Plates were coated with either phage alone, LAM, HS peptide-expressing phage, or SG peptide-expressing phage (10 μg/ml for each coating molecule) and binding by rabbit anti-LAM polyclonal antibody determined by ELISA. B. Plates were coated with BSA, LAM, HS peptide-expressing phage, HS peptide alone, HS peptide conjugated to KLH, HS scramble peptide alone (HSSR) or HSSR conjugated to KLH (10 μg/ml for each coating molecule) and binding by rabbit anti-LAM polyclonal antibody determined by ELISA. C. Plates were coated with BSA, LAM, SG peptide-expressing phage, SG peptide alone, SG peptide conjugated to KLH, SG scramble peptide alone (SGSR) or SGSR conjugated to KLH (10 μg/ml for each coating molecule) and binding by rabbit anti-LAM polyclonal antibody determined by ELISA. One of two representative experiments is shown. D. Plates were coated with BSA, LAM, HS peptide conjugated to KLH, HSSR conjugated to KLH or KLH alone and binding by serum from LAM vaccinated mice (n = 5) was tested by ELISA.

To determine if the putative mimotope peptides we identified by biopanning using the mouse monoclonal antibodies could also be detected by LAM antibodies from another species, we tested the ability of the rabbit polyclonal anti-LAM antibody to bind the putative mimotope peptides. Thus, we coated plates with LAM alone as a positive control, HS-conjugated phage, SG-conjugated phage or phage alone and determined the magnitude of binding of the rabbit anti-LAM antibody. Coating the plate with LAM yielded the greatest OD value (3.23), which was similar to coating with the HS phage (2.82) (Fig 2A). Coating wells with an equivalent amount of SG-conjugated phage yielded the lowest response (OD 1.56).

We next compared the ability of the rabbit anti-LAM polyclonal antibody to recognize the mimotope peptides under a variety of conditions. Thus, we coated plates with LAM as a positive control or HS peptide alone, HS peptide displayed on bacteriophage, HS peptide conjugated to KLH, the scrambled version of the HS peptide (HSSR) or the HSSR peptide conjugated to KLH and determined the magnitude of binding of rabbit polyclonal anti-LAM antibody to each hapten by ELISA. The OD values were 2.8, 0.45, 1.81, and 2.56 for LAM, HS peptide alone, HS phage, and HS-KLH respectively (Fig 2B). No binding was detected for the scramble peptide alone or when it was conjugated to KLH. When we repeated the experiment for the SG peptide, the OD values were 2.8, 0.32, 1.36, and 0.57 for LAM, SG peptide alone, SG phage, and SG-KLH respectively (Fig 2C). For both peptides, coating with synthetic peptide alone did not result in significant antibody binding. In contrast, while the anti-LAM polyclonal antibody bound both HS and SG peptides displayed on bacteriophage, only the HS-peptide could be bound when conjugated to KLH. Thus, we conclude that both peptides require conjugation to a larger protein to be successfully recognized by anti-LAM antibody, and that the HS peptide is more consistently detected by the anti-LAM polyclonal antibody. Because (1) the SG peptide was identified by biopanning with both monoclonal antibodies, (2) it was also identified in multiple other studies lacking a common target, and (3) it had a weaker reaction than the HS-peptide when conjugated to KLH, we were concerned that it might be a false-positive target-unrelated peptide [37] and thus focused on the HS-peptide. Finally, to determine if mice could generate anti-LAM antibodies after vaccination with LAM, and if such antibodies could detect the HS-peptide, we vaccinated outbred Swiss-Webster mice with LAM combined with alum and tested the serum against various antigens by ELISA (Fig 2D). As expected, serum from LAM vaccinated mice robustly bound LAM. Polyclonal mouse anti-LAM antibodies also bound the HS-peptide conjugated to KLH, but not the HSSR control peptide conjugated to KLH or KLH alone (Fig 2D). Thus, we conclude that anti-LAM antibodies from either rabbit (polyclonal) or mouse (monoclonal or polyclonal) bind the HS-peptide when it is conjugated to a carrier molecule.

### Mimotope activity of HS-peptide conjugated to KLH in vivo

**Fig 3:**
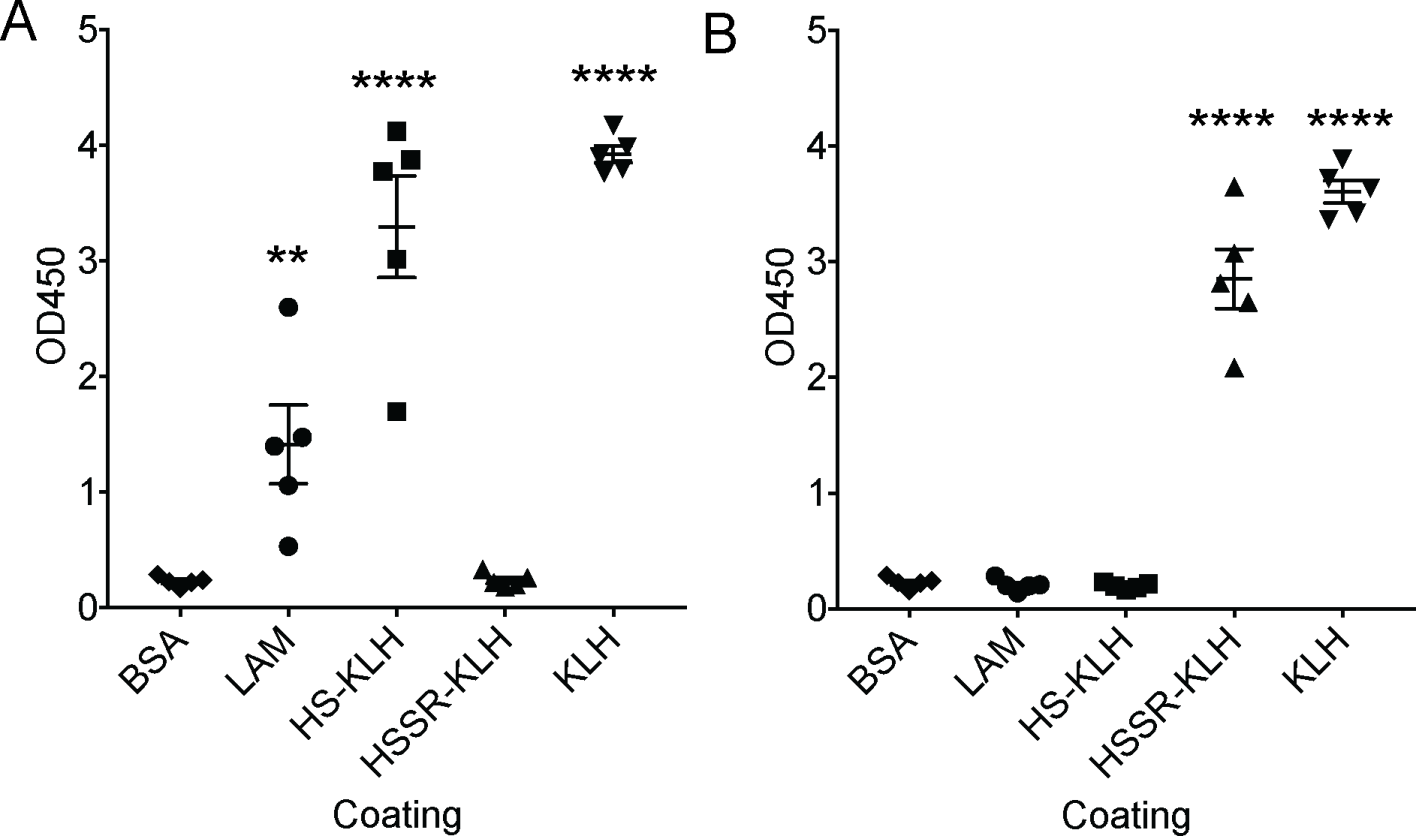
Vaccination with HS-peptide conjugated to KLH induces anti-LAM antibodies. Mice (n = 5) per group were vaccinated with HS peptide conjugated to KLH (A) or HSSR peptide conjugated to KLH (B) and sera was collected after 4 vaccinations. Sera was collected from individual mice and tested for binding (1:100 dilution) to BSA, LAM, HS peptide conjugated to KLH, HSSR conjugated to KLH or KLH alone (10 μg/ml for each coating molecule) by ELISA. Shown is a scatter plot from one experiment of two. ** p< 0.01, **** p<0.0001 compared to BSA by one-way ANOVA and Holm-Sidak test for multiple comparisons.

To confirm that the HS-peptide had mimotope activity in vivo, we vaccinated Swiss-Webster mice with KLH-conjugated HS-peptide or KLH-conjugated HSSR peptide with alum adjuvant and measured their antibody responses. After 4 injections, all mice vaccinated with the HS peptide conjugated to KLH generated antibodies against HS peptide and KLH but not the HSSR peptide or BSA, as expected (Fig 3A). Likewise, all mice vaccinated with the HSSR peptide conjugated to KLH generated antibodies against HSSR peptide and KLH but not HS peptide or BSA (Fig 3B). However, only mice vaccinated with the HS peptide conjugated to KLH developed antibodies that cross-reacted with LAM, though there was significant variation in the response of individual mice (Fig 3A). No HSSR-vaccinated mice generated antibodies reactive with LAM by ELISA (Fig 3B). Taken together, the data show that the HS peptide can function as a mimotope in vivo when conjugated to the hapten KLH in the presence of alum adjuvant.

### Peptide conjugation to baculovirus

**Fig 4:**
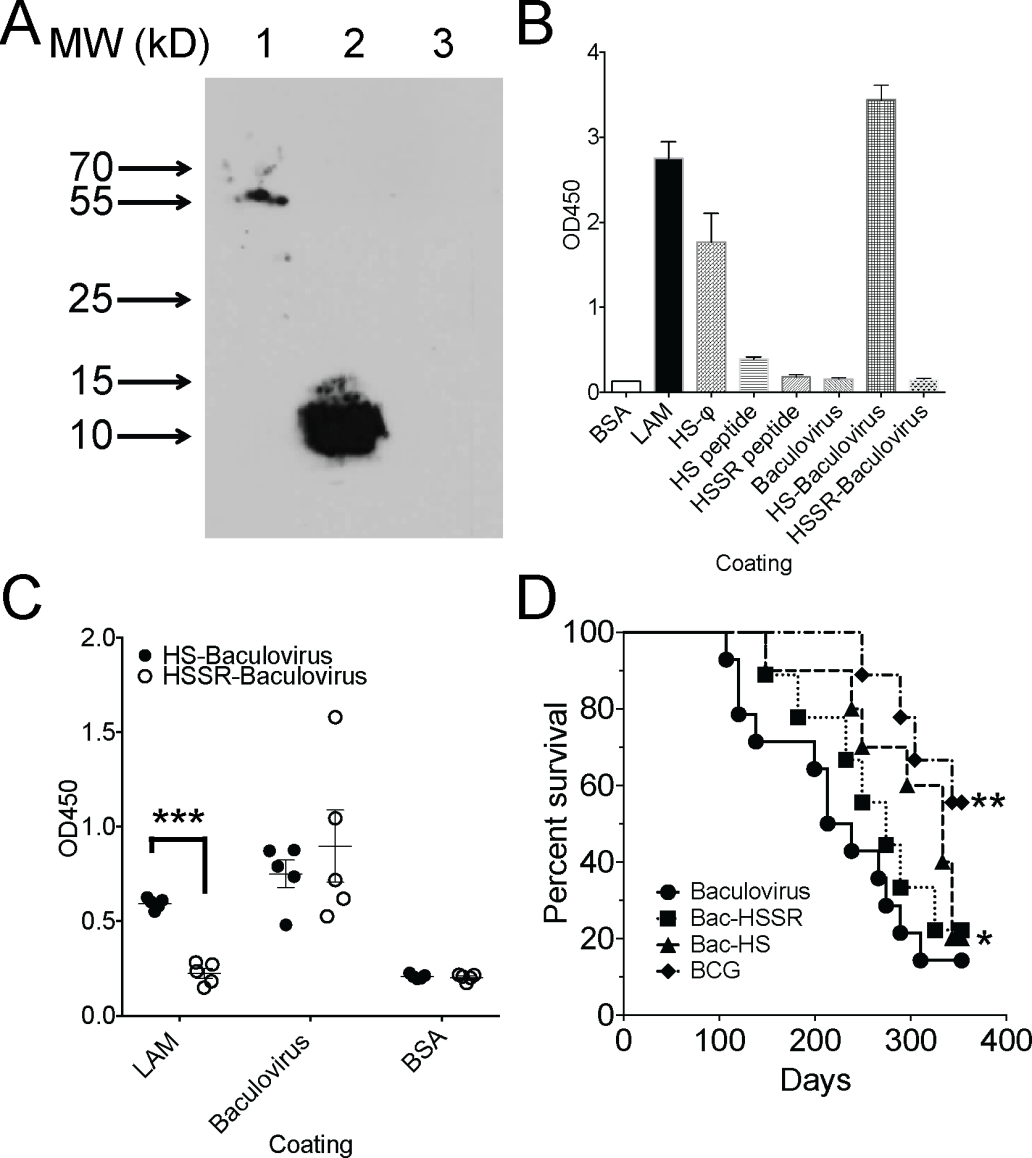
Baculovirus conjugated HS peptide is protective in a low dose aerosol tuberculosis infection model in mice. A. Test peptide (P1 biotin) was conjugated to baculovirus and detected by anti-biotin Western blot. Lane 1 is baculovirus conjugated to P1 biotin, lane 2 is peptide alone, and lane 3 is baculovirus alone. B. Plates were coated with BSA, LAM, HS peptide-expressing phage, HS peptide alone, HSSR peptide alone, baculovirus alone, HS peptide conjugated to baculovirus or HSSR conjugated to baculovirus (10 μg/ml for each coating molecule) and binding by rabbit anti-LAM polyclonal antibody determined by ELISA. C. Mice (n = 5) per group were vaccinated intranasally with HS peptide conjugated to baculovirus or HSSR peptide conjugated to baculovirus and sera was collected after 4 vaccinations. Sera was collected from individual mice and tested for binding (1:100 dilution) to BSA, LAM, or baculovirus (10 μg/ml for each coating molecule) by ELISA. Shown is a scatter plot from one experiment of two. *** p<0.005 by unpaired t-test. D. Mice (n = 10 per group) vaccinated as in C with baculovirus alone, HS peptide conjugated to baculovirus, HSSR peptide conjugated to baculovirus or M. bovis var BCG were infected with 200 CFU *M*. *tuberculosis* Erdman strain and monitored for survival. *p<0.05, **<p<0.01 by Gehan-Breslow-Wilcoxon test compared to baculovirus vaccination.

While we were in the process of biopanning for novel mimotopes, we tested if we could conjugate peptides successfully to baculovirus as has been previously reported [23]. We synthesized a biotinylated version of Peptide 1 (Table 1), conjugated it to baculovirus and then tested conjugation by Western blot and ELISA. Both peptide conjugated baculovirus (Fig 4A, lane 1) and free peptide (Fig 4A, lane 2) could be detected with an anti-biotin antibody, but baculovirus alone (Fig 4A, lane 3) could not.

### Immunogenicity of HS-peptide conjugated baculovirus

To test the ability of HS-peptide to be recognized as a LAM mimotope when conjugated to baculovirus, we coated ELISA plates with HS-peptide conjugated to baculovirus, the HSSR-peptide conjugated to baculovirus as well as controls and measured binding by ELISA using an anti-LAM antibody. As expected, the anti-LAM antibody bound LAM, the HS-peptide conjugated to bacteriophage and also the HS-peptide conjugated to baculovirus (Fig 4B). Neither baculovirus alone nor HSSR-peptide conjugated baculovirus were detected by the anti-LAM antibody, demonstrating the specificity of mimotope activity of the HS-peptide.

To further test the immunogenicity of the baculovirus conjugates as LAM mimotopes, we vaccinated mice intranasally with either HS-conjugated baculovirus or HSSR-conjugated baculovirus and evaluated the serum IgG response after 4 vaccinations. While both vaccinations yielded anti-baculovirus antibodies (Fig 4C), only vaccination with the HS-peptide conjugated baculovirus induced an anti-LAM mimotope response (Fig 4C). This mimotope activity was lower than the activity induced by KLH-conjugated HS peptide (compare Fig 4C to Fig 3A).

### Effect of vaccination on mouse survival after *M*. *tuberculosis* infection

To test the ability of HS-peptide conjugated to baculovirus to protect mice from tuberculosis, we vaccinated outbred Swiss-Webster mice with either baculovirus alone, HS-conjugated baculovirus, HSSR-conjugated baculovirus or BCG as a positive control and then infected all of the mice with 200 CFU of *M*. *tuberculosis* Erdman strain via aerosol. Compared to vaccination with baculovirus alone or baculovirus conjugated to the control scramble peptide, vaccination with the HS-conjugated baculovirus delayed *M*. *tuberculosis* associated mortality, such that at 200 days after infection, when >50% of the control animals had succumbed to disease, only 1 animal vaccinated with the HS-conjugated baculovirus had died (Fig 4D). The observed delayed survival was not associated with statistically significant differences in *M*. *tuberculosis* CFU in lung, mediastinal lymph node, liver or spleen comparing HS-peptide vaccinated mice to control mice (S1 Fig). Eventually the majority of HS-conjugated baculovirus vaccinated mice did succumb to disease. Thus, while the HS-conjugated baculovirus vaccination was partially protective, the protection was not durable and was not as robust as vaccination with BCG (Fig 4D). Taken together, this study demonstrates that an anti-LAM mimotope peptide can provide modest protection against *M*. *tuberculosis* disease.

## Discussion

Development of an improved tuberculosis vaccine has been a primary goal for decades [5]. Though many candidate gene approaches have been tried, to date none have succeeded in providing either enhanced early protection against infection or in preventing dissemination better than the currently available BCG vaccine [5]. Thus, new approaches and vaccination strategies are needed. Here we demonstrate that a peptide mimotope against the mycobacterial cell wall component LAM can be identified by panning a phage display library, that this mimotope can induce anti-LAM antibodies in mice after vaccination either when conjugated to KLH or baculovirus, and that a baculovirus-mimotope anti-LAM vaccination strategy can afford partial protection against mortality from *M*. *tuberculosis* infection in a murine model of tuberculosis.

Several previous studies have reported the development of peptide mimotopes for the mycobacterial LAM antigen (Table 1), though none of those reports tested the ability of LAM mimotopes to afford protective immunity against *M*. *tuberculosis* infection. Since we were interested in testing whether a mimotope vaccine targeting LAM would be effective during murine tuberculosis, we began by first testing the known peptide sequences. To our surprise, we were unable to detect mimotope activity for the published peptide sequences, neither in vitro as antigenic substrates in ELISA nor in vivo after vaccinating experimental animals. One possible explanation, at least in the case of the Gevorkian study [29] is a difference in antibodies used for phage display and detection of mimotope activity. Gevorkian used lab-generated rabbit polyclonal antibodies against neutral polysaccharides for selection of phage clones, which were then tested for LAM mimotope activity [29], while we used monoclonal antibodies CS-35 and CS-40. For the other two studies, technical differences in performing ELISAs might account for the disparate results.

We screened a phage display library to identify peptide mimotopes targeting LAM on *M*. *tuberculosis*. After multiple rounds of enrichment only one peptide, detected by the CS-40 mAb, demonstrated robust and specific mimotope activity both in vitro and in vivo after vaccination of experimental animals. Interestingly, the peptide we identified was also enriched in a phage display screen for peptides bound by the insulin-like growth factor 2 receptor (IGF2R), which is also known as the cation-independent mannose-6-phosphate receptor (M6PR) [40]. Thus, since LAM is composed of a number of mannose residues, the CS-40 mAb is specific for ManLAM, and the M6PR contains multiple mannose-6-phosphate binding domains [45, 46], it is possible that in vivo the HS peptide creates a three-dimensional structure similar to the mannose component of ManLAM, thus leading to a mimotope response. Indeed, ManLAM is bound by the M6PR, where it mediates adherence to and uptake of *M*. *tuberculosis* by human macrophages [47, 48]. However, once *M*. *tuberculosis* is ingested it excludes the M6PR from its surrounding phagosome [49] likely via ManLAM-mediated inhibition of phagosome maturation [50].

In an effort to develop a mucosal approach for vaccination against *M*. *tuberculosis*, we took advantage of a relatively understudied approach, namely, to conjugate a peptide mimotope to baculovirus and to administer this vaccine intranasally. While this approach successfully induced an IgG mimotope response against LAM as detected by ELISA, the mimotope response with baculovirus vaccination was not as robust as the response obtained by the more conventional method of vaccination with a KLH-peptide molecule. There are several possible explanations for this difference, including (i) the possibility that there are more available lysine residues for peptide conjugation on KLH as compared to the surface of baculovirus, thus increasing the number of mimotope peptide administered, (ii) that KLH itself might be more immunogenic than baculovirus, (iii) that the subcutaneous route of administration for the KLH-peptide conjugate is more potent than the intranasal route for baculovirus and finally (iv) that the additional administration of the alum adjuvant with the KLH-peptide conjugate enhanced the immune response as compared to the baculovirus-peptide conjugate alone.

Despite the relatively modest mimotope response, vaccination with the HS-baculovirus did afford mice some protection against *M*. *tuberculosis* mortality, though not as effectively as the established BCG vaccine. One possible explanation for this observation is that development of anti-baculovirus antibodies during the vaccination process, which we observed in this study, may have reduced the overall efficacy of the intranasal baculovirus vaccination strategy compared to BCG alone by reducing the effectiveness of booster vaccinations. An alternative explanation is that serum anti-LAM antibodies alone are not sufficient to protect mice from tuberculosis infection, and that antibodies against additional cell wall components might improve the overall protection. To that end, identification of mimotopes against other important cell wall antigens might provide even greater protection when combined with anti-LAM mimotopes. *M*. *tuberculosis* has an extraordinarily complex cell wall with multiple potential immunogenic targets beyond LAM such as mycolic acids, peptidoglycan, arabinomannan (AM), lipomannan (LM), and phosphatidylinositol (PI) mannosides (PIMs), and the lipids phthiocerol dimycocerosate (PDIM), sulfolipid-1 (SL-1), and phenolic glycolipid (PGL) [51, 52]. Indeed, vaccination of either guinea pigs [53] or mice [54] with mycobacterial AM conjugated to immunogenic peptides affords protection from tuberculosis-induced mortality and dissemination during experimental infection. Antibodies against either AM [55] or LAM [56] are detectable in the serum of *M*. *tuberculosis* infected humans, and antibodies against AM develop in the setting of BCG vaccination of humans [9], indicating that the molecules themselves are immunogenic during infection. Ultimately, combining peptide mimotopes against multiple cell wall components may enhance the efficacy of a tuberculosis vaccine, whether they are conjugated to baculovirus or other carriers in combination with a potent adjuvant. Such a mimotope vaccine could also include whole polypeptides representing immunodominant antigens so as to create an additive or synergistic immune response.

## Supporting information

S1 Fig *M. tuberculosis* CFU in mice after vaccination with mimotope conjugated baculovirus, controls or BCG. Vaccinated mice (n = 5 per group) were infected with *M*. *tuberculosis* and 30 days after infection, lung, mediastinal lymph nodes, liver and spleen harvested for CFU enumeration. Shown for each group is the geometric mean with geometric standard deviation.
(TIF)
