# Supplementary figures and images for: A baculovirus-conjugated mimotope vaccine targeting Mycobacterium tuberculosis lipoarabinomannan
Source: PLoS One. 2017 Oct 5;12(10):e0185945. doi: 10.1371/journal.pone.0185945 (PMC5628901; doi:10.1371/journal.pone.0185945)

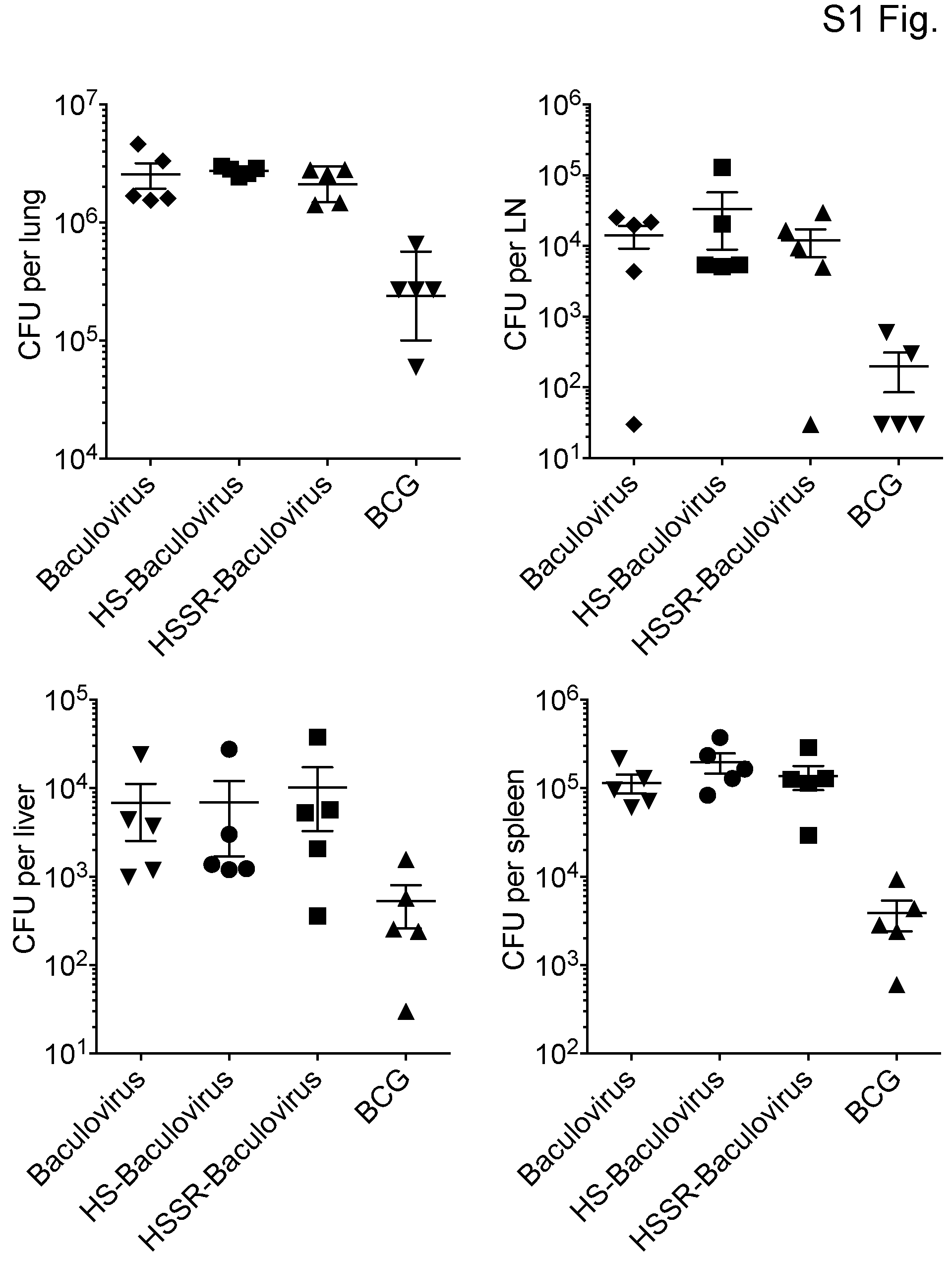

Supplement: S1 Fig — Vaccinated mice (n = 5 per group) were infected with M. tuberculosis and 30 days after infection, lung, mediastinal lymph nodes, liver and spleen harvested for CFU enumeration. Shown for each group is the geometric mean with geometric standard deviation. (TIF) [file pone.0185945.s001.tif]
